# Influence of simulation and clinical settings on peripheral vein cannulation skill learning in nursing education: A qualitative study

**DOI:** 10.1016/j.ijnsa.2023.100123

**Published:** 2023-03-20

**Authors:** Monika Ravik, Ida Torunn Bjørk

**Affiliations:** a Department of Nursing and Health Sciences, Faculty of Health and Social Sciences, University of South-Eastern Norway, Post-box 235, 3603 Kongsberg, Norway; b Department of Public Health Science, Faculty of Medicine, University of Oslo, PB 1089 Blindern, 0318 Oslo, Norway

**Keywords:** Ad hoc conversations, Clinical setting-based skill learning, Focus group interviews, Peripheral vein cannula, Peripheral vein cannulation, Simulation-based skill learning, Transfer of learning

## Abstract

**Background:**

Peripheral vein cannulation is a complex yet common practical skill. Learning to insert a peripheral vein cannula is fundamental in nursing education; however, the most beneficial pedagogical approaches are yet to be elucidated.

**Objective:**

To explore and impart a deeper understanding of the learning conditions in nursing education for developing competency in peripheral vein cannulation.

**Design:**

Qualitative, explorative. and comparative research design

**Setting(s):**

Two nursing educational settings in southern Norway: an academic setting for simulation-based peripheral vein cannulation skill learning, followed by a hospital setting that provided a 9 week clinical placement period.

**Participants:**

Nine student nurses in the second year of a bachelor's programme in nursing.

**Methods:**

Focus group interviews, individual interviews, and ad hoc conversations with the student nurses on their experiences during and after the process of developing competency in peripheral vein cannulation. Thematic analysis was used to identify categories and common themes.

**Results:**

Eight major themes were identified: ‘Anatomical and physiological conditions related to the training modalities’, ‘Realism in training’, ‘Sequences in peripheral vein cannulation training’, ‘Different training modalities affording varied learning opportunities’, ‘Professional nursing assessments’, ‘Patients’ and peers’ emotional reactions’, ‘Student nurses’ own emotional reactions’, and ‘Significance of the relationship between the student nurse and patient’.

**Conclusions:**

Simulation-based peripheral vein cannulation practice was an important starting point for the students’ skill learning. However, the students experienced the complexity of the skill only in the clinical setting because it offered several learning opportunities. Nonetheless, our findings indicate a need to further review peripheral vein cannulation skill learning, especially patient contributing factors, to enhance the transfer of learning from the simulation setting to the clinical setting.

**Tweetable abstract:**

Clinical setting-based peripheral vein cannulation practice is vital for student nurses’ skill learning because of the skill's complexity

## 1 Background

Peripheral vein cannulation is a complex practical skill (Gregersen et al., 2021; Liou et al., 2020; Massey et al., 2020), involving psychomotor aspects, ethical considerations, theoretical and practical knowledge, and communication and relational aspects (Bjørk and Kirkevold, 2000). It is a vital practical nursing skill (Alexandrou et al., 2018; Hunter et al., 2018; Morgaonkar et al., 2017) and is a common invasive medical skill performed by both registered nurses (Etafa et al., 2020; Parker et al., 2017; Massey et al., 2020; Wong et al., 2018) and medical doctors (Breindahl et al., 2023). Vein cannulation provides access to the patient's circulatory system via a plastic-sheathed cannula for essential medical treatment, such as administration of drugs, blood, and intravenous fluids and withdrawal of blood samples (Larsen et al., 2022; Wong, 2018).

In the clinical setting, inserting a venous cannula must be expeditious for initiation of medical treatment. Many registered nurses fail in their attempted vein cannulations (Choden et al., 2019; Cooke et al., 2018; Piper et al., 2018). Nurse educators should assess and audit educational strategies employed to enhance student nurses’ peripheral vein cannulation skill learning (Garcia-Expòsito et al., 2021), which remains insufficiently explored in nursing education (Hilleren et al., 2022).

Often student nurses learn practical skills, including peripheral vein cannulation, in two settings – simulation and clinical (Hayden et al., 2014; Hilleren et al., 2022; Rajaguru and Park, 2021). Simulation is a promising approach that allows learning in a structured, low-stress context without adverse consequences for actual patients (Bridge et al., 2022; Hayden et al., 2014; Rajaguru and Park, 2021; Valizadeh et al., 2021). Moreover, simulation-based peripheral vein cannulation skill learning allows students to undertake repeated attempts of the same practical skill and to learn from their mistakes (Arslan, 2021). In general, practical nursing skills are learned and practiced on partial-task trainers and peers in preparation for clinical learning experiences (Bridge et al., 2022; Arslan, 2021; Valizadeh et al., 2021). However, practice exercises for such invasive skills are not included in all nursing education strategies (Hilton and Barrett, 2009).

Partial-task trainers are somewhat disadvantageous in that the user interface is passive, and vein cannulation is practiced with minimal response from the simulator (Bridge et al., 2022; Ravik et al., 2015). Simulation-based peripheral vein cannulation skill learning is often a technical endeavour wherein the aim is the successful insertion of the cannula into the vein (Ravik et al., 2015; Ravik et al., 2017a). This can be defined as a mechanistic approach to learning a fundamental nursing skill (Kitson et al., 2014). When learning peripheral vein cannulation, students often encounter partial-task trainers with visible veins (Reinhardt et al., 2012) and peers with veins that are uninfluenced by diseases (Lund et al., 2012). The role of simulation-based peripheral vein cannulation skill learning in nursing education remains unclear because the influence of this training modality on students’ learning outcomes is not yet fully understood (Arslan, 2021; Hilleren et al., 2022; Wang et al., 2022).

Unlike simulation-based learning, clinical skill learning occurs in a complex learning environment that involves actual patients (Larsen et al., 2022; Matchim and Kongsuwan, 2015). Thus, students have the opportunity to practice vein cannulation in a setting where their technical achievements need to be integrated with psychosocial and relational aspects of care. Patients have medical disorders that cannot be replicated by a simulator or peer, and these provide students with more complex learning opportunities than those offered in simulation-based learning (Larsen et al., 2022; Matchim and Kongsuwan, 2015); for example, a patient's veins are often invisible and not palpable or easy to perforate due to various illnesses (Gjerde et al., 2021; Jacobson and Winslow, 2005).

Furthermore, students should communicate with the patient and consider the patient's experience of the situation, such as stress or anxiety (De Fazio et al., 2017). A lack of mastery of vein cannulation may cause students to become passive observers in the learning situation (Ravik et al., 2017a). Hence, a more contextual understanding beyond the technical and instrumental aspects of peripheral vein cannula insertion is required (Alexandrou et al., 2018). Therefore, an understanding of patient-contributed learning conditions is essential when students practice vein cannulation in the clinical setting.

### 1.1 Theoretical perspective

We were inspired by Marton's (2006) theory of transfer of learning. Marton (2006) defined transfer of learning as the adaptation of learning and knowledge from one situation to another. A connection (similarities) between learning and application tasks, situations, or settings is vital for this transfer, thereby ensuring that the learning from one situation can be perceived, reinterpreted, and applied in another. Additionally, Marton (2006) described that discerning critical and small differences between the learning and application situations is beneficial because the learner is able to adjust to new situations. Without this focus, a learner has no opportunity to discern elements of the subject to be learned, and transfer of learning will be restricted (Marton, 2006).

### 1.2 Aim and research question

This study aimed to impart a deeper understanding of the learning conditions for developing peripheral vein cannulation competency. We explored the student nurses’ experience of peripheral vein cannulation skill learning using different learning modalities: latex arms (partial-task trainers), peers, and patients and evaluated what influenced their cannulation skill learning.

The following research question was postulated:

‘What influences student nurses’ peripheral vein cannulation skill learning as they interact with latex arms, peers, or patients?’

## 2 Method

### 2.1 Design

This study is a part of a larger study exploring practical skill learning in nursing education in the simulation- and clinical settings (Ravik et al., 2017a; Ravik et al., 2017b; Ravik and Bjørk, 2021) and transfer of skill learning from the simulation setting to the clinical setting (Ravik et al., 2015). This study had a qualitative, explorative, and comparative research design with a data-driven inductive approach that went beyond descriptions to provide the researchers with an in-depth understanding of peripheral vein cannulation skill learning (Kvale and Brinkmann, 2015; Polit and Beck, 2020).

### 2.2 Study sample and recruitment

Participants were second year-student nurses of a bachelor's programme in nursing from southern Norway. Ninety-three full-time students were invited to participate in the study; 27 consented; and finally, nine students were drawn from this group. We considered that nine students were enough to create saturation because a great amount of data was collected from each student based on a variety of interviews. In addition, nine students were a practical number in the simulation setting as this provided three groups with three students in each group who trained together and talked about the same situations from different approaches. These students were purposely selected (Patton, 1990), and the inclusion criteria were first clinical placement in a hospital, no previous experience with peripheral vein cannulation, and no prior hospital experiences anywhere.

### 2.3 Study settings

The study was conducted in two nursing education settings: an academic setting after simulation-based peripheral vein cannulation skill learning, followed by a hospital setting that was a 9 week clinical placement period. The students were free to choose whether they wanted to practice on a latex arm or a peer's arm (on the dorsum of the hand). These choices are shown in Table 1. The students were supervised by an educator with approximately 20 years of experience of supervision in simulation-based learning. The students were placed in various medical units, and they practiced peripheral vein cannulation on the dorsum of the hand or forearm of patients in the clinical setting with acute, critical, or chronic illnesses related to lung diseases, neurological disorders, infection, or cancer. Patients were 16 years and older. The students were individually supervised by a registered nurse. Table 1 presents the number of peripheral vein cannulation attempts of the students in the clinical settings. Detailed information about video-recorded peripheral vein cannulation attempts has been described in previous studies; namely, Ravik et al., 2015; Ravik et al., 2017a and Ravik and Bjørk, 2021.

**Table 1:** Training modalities and opportunities to practice peripheral vein cannulation.

Student	Simulation centre latex arm	Simulation centre peer	Clinical setting, the number of peripheral vein cannulation attempts on patients
1	1	0	12 (3 failures). Video-recorded at 5 attempts
2	1	1	5 (4 failures). Video-recorded at 3 attempts
3	1	1	11 (5 failures). Video-recorded at 3 attempts
4	1	0	11 (9 failures). Video-recorded at 5 attempts
5	0	2	8 (4 failures). Video-recorded at 4 attempts
6	0	2	8 (4 failures). Video-recorded at 4 attempts
7	2	0	9 (5 failures). Video-recorded at 5 attempts
8	0	2	13 (11 failures). Video-recorded at 6 attempts
9	2	0	4 (4 failures). Video-recorded at 3 attempts

### 2.4 Data collection

Triangulation of qualitative data collection methods was used. Focus group and individual interviews and ad hoc conversations were used to secure a comprehensive understanding of the phenomenon of interest from different perspectives (Patton, 1990) (Table 2).

**Table 2:** Overview of the data collection methods and magnitude.

Student	Focus group interview	Individual interviews	Ad hoc conversations
1	1	2	8
2	1	2	5
3	1	2	5
4	1	2	5
5	1	2	5
6	1	2	5
7	1	2	5
8	1	2	7
9	0	2	0

Two semi-structured focus group interviews were conducted immediately after the simulation-based peripheral vein cannulation skill learning with three and five students, respectively (one of the students was prevented from participating in the focus group interview due to transportation problems). Focus group interviews were conducted to generate rich discussions from participants with similar backgrounds, though with varying perceptions and reactions (Polit and Beck, 2020). In the academic setting, both focus group interviews were conducted in a secluded room for a total of approximately 60 min. The interviewer initiated each focus group interview by presenting the objectives and asking participants to share their experiences and thoughts throughout the interview. The time was not distributed among the participants, but everyone was encouraged to be active and engaged throughout the interview situation.

Individual interviews provided insight from participants’ own experiences, and some participants may have felt more comfortable sharing their experiences in the presence of fewer listeners than others (Kvale and Brinkmann, 2015; Polit and Beck, 2020). In the hospital setting, 18 semi-structured individual interviews were conducted in a protected room, undisturbed, for 60–90 min each. Each student was individually interviewed twice (sequentially). The first and second individual interviews were conducted during the first and last week of clinical placement, respectively. The last individual interview was based on video-stimulated recall. This technique involved interviewing the participants as they viewed video-recorded segments of their own behaviour (Gazdag et al., 2019).

Semi-structured interview guides were developed to direct both types of the interviews and ensure coverage of relevant areas in the discussions (Polit and Beck, 2020). The focus of the interviews is summarised in Table 3.

Additionally, in the clinical setting, 45 ad hoc conversations or informal-toned spontaneous communications (Groenland and Dana, 2019) were conducted in the storage rooms at the units. These conversations were conducted whenever possible, before and after the students’ peripheral vein cannulation attempts, without the use of a formal interview guide and were approximately 5–25 min in duration. The interviews and ad hoc conversations were audio-recorded and transcribed verbatim. The focus of the conversations is summarised in Table 3.

**Table 3:** Focus of the three interviews and ad hoc conversations with examples of questions.

Focus group interview	First individual interview	Second individual interview	Ad hoc conversations
The focus was directed toward simulation-based peripheral vein cannulation skill learning, though with a link directed toward thoughts about the imminent performance of peripheral vein cannulation in the clinical setting.	The focus was directed toward practicing peripheral vein cannulation on a patient immediately after simulation-based learning, how the students prepared for their practice, and whether and how simulation-based training prepared them for practicing peripheral vein cannulation in the clinical setting.	The focus was directed toward learning and practicing peripheral vein cannulation on patients during the students’ clinical placement, and how they, at the end of their 9 weeks in the clinical setting, viewed simulation as a preparatory learning strategy.	The focus was directed toward thoughts about practicing peripheral vein cannulation on ‘patient X’ (immediately before peripheral vein cannulation) and experiences toward practicing peripheral vein cannulation on ‘patient X’ (immediately after peripheral vein cannulation.
Example of an interview question: Can you describe positive experiences related to the simulation-based training and learning of peripheral vein cannulation?	Example of an interview question: How did you experience that the simulation-based training and learning of peripheral vein cannulation influenced your performance of peripheral vein cannulation on patients in the clinical placement setting?	Example of an interview question: Did you experience any particular challenges when you performed the practical skill of peripheral vein cannulation during the clinical placement period? Please describe.	Example of a question related to ad hoc conversations: How did you experience aspects of the currently performed attempt of peripheral vein cannulation?

### 2.5 Data analysis

Data from the interviews and conversations were merged, with a focus on the study's aim. Kvale and Brinkmann (2015) principles of interpretative analysis guided the inductive analysis of the data. Both authors independently and collaboratively performed the analysis and repeatedly revisited the transcribed interviews during the analysis. The analysis involved three levels of interpretation: self-, critical common sense, and theoretical understandings. In self-understanding, researchers interpreted a condensed form of the interviewed participants’ own understanding of the meaning of their statements. The meaning units were extracted after repeatedly reading the transcribed interviews and ad hoc conversations, and these were formulated in codes that preserved the participants’ own ways of expression. Critical common sense understanding involved using a wider frame of understanding during interpretation based on researchers’ insight into the research theme. The codes were collapsed into sub-categories and subsequently into more overarching categories and themes. Sub-categories, categories, and themes were formulated in a professional language excluding the participants’ self-understanding. Quotations from the transcribed text strengthened and exemplified this level of interpretation. In theoretical understanding, interpretation involved the use of relevant theory.

### 2.6 Rigour

Lincoln and Guba (1985) proposed four criteria to ensure study trustworthiness: credibility, dependability, transferability, and confirmability. Triangulation of data collection methods was used to enhance data completeness. Both authors repeatedly read and discussed the analysis process and data content to ensure the credibility of the analysis. The use of interview guides was particularly important to ensure consistent data collection. The interviews were carefully transcribed and checked for accuracy. Both authors performed the coding and analysis processes. Dependability and transferability were established by quoting participants’ actual statements. The transcripts were continually referred to during the data analysis. Broad descriptions were provided for the assessment of transferability. Confirmability was ensured by detailed and stepwise description of the data collection and analysis processes (Lincoln and Guba, 1985).

### 2.7 Ethical considerations

Institutional and unit permissions were obtained, and the research was conducted in accordance with the Declaration of Helsinki (World Medical Association, 2013). The study was approved by the Norwegian Social Science Data Service (project number 30181). The students’ participation in the study was voluntary; written informed consent was obtained from all participants. Confidentiality, anonymity, and the right to withdraw participation at any time were guaranteed. Participants were assured that participation would not influence the evaluation of their progress during clinical placement. Student-signed consent forms, audio recordings, and transcribed text were kept in a password-protected computer.

To protect participants’ anonymity, the educational setting and student characteristics are not disclosed in the method or study results.

## 3 Results

Eight themes pertaining to the study's aim were identified in the analysis: ‘Anatomical and physiological conditions related to the training modalities’, ‘Realism in training’, ‘Sequences in peripheral vein cannulation training’, ‘Different training modalities affording varied learning opportunities’, ‘Students’ own emotional reactions’, ‘Patients’ and peers’ emotional reactions’**, ‘**Significance of the relationship between the student and patient’, and ‘Professional nursing assessments’.

### 3.1 Anatomical and physiological conditions related to the training modalities

The students’ experience of peripheral vein cannulation practice on latex versus human arms was vastly different. Patients’ veins were often described as being thin, stiff, rolling, and easily ruptured, whereas a latex arm's veins were inflexible. Moreover, the latex arm's veins were constantly visible, whereas in human arm veins, visibility was location-dependent.

*That vein on a human was very deep and therefore difficult to assess (Student 8).*

A latex arm was described as being stiff and with inelastic ‘skin’, whereas a peer's arm had soft, elastic, and whole skin. In a hospital setting, the students experienced that the skin on patient arms was considerably different. It varied between being thick and thin; it could be brown, bruised, affected by previous drug treatment, or had multiple scars post and prior to peripheral vein cannulation attempts.

*The skin had a lot of scars, I thought the patient had injured herself because it looked like she had cut herself, but the reason was just that the patient had a steroid-damaged skin (Student 2).*

The cannula had to be forced through the ‘skin’ and ‘vein’ on a latex arm, and the cannulation could be performed in one movement.

*You have to stick the cannula so hard into the latex arm to get through it (FG interview 2).*

Conversely, minimal force had to be used when the students penetrated the vein on a human arm. It was a step-by-step process; the skin and vein had to be penetrated before the cannula could be further inserted into the vein.

*Moving the cannula through skin and vein was like taking it through paper, it was really easy, I thought I would feel a kind of resistance, but it was suddenly through the vein on the other side (FG interview 1).*

### 3.2 Realism in training

The students used the same type of equipment regardless of the training modality. This made the students familiar with the equipment, and similarities contributed to realism in the training situation.

The students concurred that peripheral vein cannulation practice on a latex arm limited realism in the training situation and appeared to be somewhat unrealistic.

*I could not look the latex arm in the eyes because it didn't have a face (Student 9).*

Furthermore, when the students practiced on a latex arm, they often pretended that the steps of the skill were performed. Consequently, the students became less serious and focused in their training.

*We get very unstructured when we pretend because we end up talking about other things and scrolling on the phone (FG interview 1).*

The students found the training situation to be more realistic and serious when they practiced vein cannulation on each other's arms. They were anxious and apprehensive of injuring their peers. However, although the students were concerned and responsible, this modality was less realistic than practicing on patients in the clinical setting.

*By all means, I was careful, but it became a bit silly and we joked. It was not present enough in my mind that I practiced vein cannulation on a human (peer). I was not serious enough. Do not misunderstand me, but it is, it is different when it is a peer rather than a patient (Student 8).*

The realism and complexity of peripheral vein cannulation was particularly experienced in the clinical setting.

*The patient had suffered from a stroke, and all the stroke patients receive blood thinning medication, and they often bleed much more easily. I did not actually think about it, but it was maybe the reason why there was so much blood flowing from her arm. I should have thought about it when I performed the skill (Student 4).*

### 3.3 Sequences in peripheral vein cannulation training

The students expressed that they had respect for peripheral vein cannulation performance skills. A step-by-step approach in practice was valuable; namely, practice on a latex arm, followed by a peer's arm, and finally, a patient's arm. Familiarity with the different steps of the skill performance such as practicing on a latex arm before progressing to a peer's arm was vital.

*It is a barrier to break the skin of another person's skin, but I felt like it was going to be okay after first trying on a latex arm. To penetrate the skin and vein on a latex arm before penetrating the skin and vein on a peer's arm provided comfort, and I thereafter believed that I would master the skill well (FG interview 1).*

Students felt mentally prepared to practice vein cannulation on a patient's arm after practicing it on a peer's arm in the simulation setting.

*I thought to myself that I have done this before. I did it, it is good. I know what I am doing (Student 1).*

For the students who had practiced vein cannulation on only a latex arm before practicing it on a patient's arm, mastering the skill in the clinical setting was a challenge.

*The students who trained on each other's arms in the simulation setting mastered the skill performance on patients much better than I did, who only trained on a latex arm. I had to practice the vein cannulation several more times on patients than the other students to master the skill performance (Student 7).*

Practicing peripheral vein cannulation on peers in the simulation setting was crucial for students to swiftly begin practicing the skill on patients in the clinical setting.

*First, I practiced vein cannulation on a latex arm in the simulation setting. If I had practiced using only this training modality, I would probably have observed a registered nurse performing the skill on a patient before I had tried to do it. Additionally, I would have wanted a lot of supervision from the nurse when I practiced the skill. But the day after I practiced on a latex arm, I practiced the skill on a peer's arm. Then, I felt ready to practice the skill on a patient in the clinical setting (Student 3).*

### 3.4 Different training modalities afford varied learning opportunities

Practicing vein cannulation on both a latex and peer's arms in the simulation setting provided students with different learning opportunities. Although students who practiced on a latex arm expressed uncertainty about the skill performance, it did not hinder them from further practice.

*‘Just jumped right into it when I practiced on a latex arm’ (FG interview 2).*

Making mistakes on a latex arm was considered acceptable and as part of the preparatory practice for vein cannulation on a peer's arm.

*Finding the grip on the vein cannula and learning from mistakes when practicing on a latex arm is desirable before I practice peripheral vein cannulation on a human (FG interview 1).*

The students expressed that they could not recall the different steps of the skill practice on a latex arm; however, they were more keen and focused when they practiced vein cannulation on a peer's arm.

*If I had practiced on a human arm, I would have remembered to take off the tourniquet. When I practice on a latex arm, I do not think about the discomfort the ‘patient’ might experience because of the tourniquet being still attached (FG interview 2).*

Erring during vein cannulation on a peer's arm resulted in serious consequences for students’ skill practice. The task had to be terminated, and the students lost opportunities to practice the final steps.

*The vein cannulation had to be terminated several times when I practiced on a peer's arm because blood in the flashback chamber was missing (FG interview 2).*

Some students stated that they had opportunities for repetitive skill practice in the clinical setting.

*Get more drilled in the clinical setting, I know how to drag the skin (Student 4).*

### 3.5 Students’ own emotional reactions

Penetrating another person's skin was a major barrier in the students’ practice. They used several emotionally charged expressions when describing their first experiences of practicing peripheral vein cannulation on a peer.

*It was deathly horrible and scary to penetrate the skin on my peer in the simulation centre (FG interview 1).*

*It is disgusting to stick a cannula in your peer's arm – especially because you know her (Student 2).*

Knowing the peer and experiencing their nervousness and observation by peers increased students’ apprehension.

Another major barrier was fear or worry of inflicting pain during cannulation when the students practiced on patients.

*I must learn to do it faster. When I am slow and sort of coax the cannula into the skin and vein, I hurt the patient. I have to practice, practice, and practice (Student 4).*

The students acknowledged that some patients were already in pain, and that their fumbling with the cannula, vein perforation, and repeated cannulation attempts probably increased the patients’ pain.

*Stabbing their skin – I am very nervous because I know they are sick, and I do not want to cause more harm. I think it is rather rude to ask for permission to stab with a cannula, and when you miss the vein, you just say: oh, I am sorry. It is not kind (Student 9).*

Despite their apprehension about skin penetration, students were happy that they could begin practicing peripheral vein cannulation on a peer.

*To get the feel of it - and that it is not a crisis to hold on to the cannula, and you do not pass out or die when someone stabs you (FG interview 1).*

First-time failure on a peer was less frightening than failure on a patient, and some students acknowledged that a cannulation on their own arm prepared them before attempting it on a patient. Students who practiced on a latex arm in the simulation centre expressed that it gave them a feeling of security, although it was considerably different from performing peripheral vein cannulation on a patient.

*The only thing I could think when I tried on a patient the first time was that I regretted enormously that I had not tried on a peer in the simulation centre. The plastic arm is something quite different. But I was so stubborn then. I did not want to try on a student's arm. In fact, I was actually quite shocked by the difference between a plastic arm and a human arm (Student 7).*

Many students described patients’ influence on their performance. Nervous patients made them more edgy, calm patients were reassuring, and involved patients made them feel safer.

*The patient was active and nice. He took care that I did it the right way. Not in an embarrassing way, just so I felt safe (Student 1).*

One student was grateful when a patient with thin veins suggested that she wait and watch while the nurse performed the peripheral vein cannulation.

### 3.6 Patients’ and peers’ emotional reactions

Students were markedly less preoccupied with the emotional reactions of their peers during peripheral vein cannulation practice than when practicing on a patient. They checked their peers’ facial expressions when applying the tourniquet or performing the cannulation and mentioned that discomfort was the main reaction.

*We have to look up all the time to see if it hurts (FG interview 1).*

A student mentioned that one peer refused to let her perform the cannulation on her, whereas another was extremely nervous.

*I penetrated her skin, and she was so nervous that she just screamed and sat with her eyes shut (Student 2).*

When expressing their views on the patients’ emotional reactions, the students were often surprised that many patients trivialised this particular practical skill, as some students were certain that all patients would be nervous to receive a peripheral vein cannula. Receiving a vein cannula appeared insignificant for some patients.

*They do not care about the skin and vein perforation or a wobbly cannula – they care about their illness (Student 3).*

Several students commented on patients’ senses of humour. Such patients were more tolerant if the students’ erred, and humour could reduce a tense situation. Students clearly expressed that they did not want to inflict pain; nevertheless, several patients expressed discomfort during peripheral vein cannulation.

*I think it is unpleasant for the patient when you have to try again. I know myself that it is no fun to feel that cannula twice (FG interview 2).*

One student felt pressured to perform peripheral vein cannulation faster than she was capable of because she felt that the patient did not want a student to practice on her. This corresponds to another student's comment on the need to interpret the patient's often conflicting signals, regarding the acceptance of the student's performance of the skill. Creating a secure setting for the patient was the ultimate aim.

*It is important not to acquire bad habits. It has to be safe for the patients – everything needs to be kept sterile (Student 4).*

After practicing peripheral vein cannulation in the clinical setting, the students commented on the variation in reactions between performing on latex arms and patients.

*You must*make *small talk with the person in the clinical setting and ensure he is not scared and that he feels taken care of. This is quite different from practicing on a plastic arm that has no feelings or thoughts. The mental thing - that is the greatest difference (Student 1).*

The students commented that peers were more nervous than patients and ‘made a lot of fuss’, which influenced the realism of the performance. However, peers could say ‘no’, while patients had no choice; they had to receive a peripheral vein cannulation for their medical treatment.

### 3.7 Significance of the relationship between the student and patient

The students acknowledged that they sometimes performed peripheral vein cannulation on patients with whom they were not acquainted. This was rather common, though not optimal. Knowing the patient could alleviate a tensed situation.

*I prefer doing the peripheral vein cannulation on a patient in my group. We have met and introduced ourselves. I know the patient, and we have started creating a nurse-patient relationship (Student 3).*

Communicating with the patient was an essential part of skill performance. Irrespective of whether the cannulation went well, talking to the patient was necessary and an essential element. Moreover, speaking clearly and slowly to patients who were old or a bit confused was crucial.

*I am even more conscious not to offend the patient when he does not know or is not quite aware of what is going on (Student 8).*

It was easier if the patient wanted to enter into a conversation for small talk. The students’ own ways of communicating were in focus as well.

*Every patient is different. Putting in the cannula is the same, but how you act varies. Sometimes I use more time to calm the patient or maybe you need to talk more than just to alert the patient when you stick the cannula into the skin. I think it is fun to meet such patients (Student 3).*

Cooperation and learning were the key terms that students used to characterise their relationship with patients. Many patients took the initiative or were willing to be involved when the students asked them about their experience with prior peripheral vein cannulations.

*…and then the patient said, just remember this, and remember that, and just take it easy and be calm. He helped me plan the procedure (Student 2).*

The students were aware that patients were experts on their own veins and that many had prior peripheral vein cannulation experience.

*I had brought the blue cannula, and the patient said he used the pink one. So, I just did what he said because he is here every third month and has a lot of experience with previous cannulations (Student 6).*

### 3.8 Professional nursing assessments

The students experienced the need for professional nursing assessments to decide on vein cannulation for patients in the clinical setting. These could be assessments related to physical limitations in the patient, medical conditions due to the patient's illness, or students’ own knowledge and competency. In general, the students had a strong focus on the patient's situation. They observed and assessed patient reactions and the actions that could benefit the patient during vein cannulation.

*I try to keep a focus on the patient, to conduct a dialogue, and be gentle in what I do, I am very focussed (Student 6).*

Assessments regarding physical limitations could occur when a vein cannula could not be inserted in the upper limb and had to be inserted in the lower limb, contrary to the students’ previous learning and practice in the simulation setting.

*I have only learned that there is a lot of risks in inserting a vein cannula into a leg (Student 3).*

The students assessed that an appropriate cannula angle to be of particular importance to master peripheral vein cannulation in patients.

*In the simulation setting we learned to angle the cannula quite high when penetrating the skin, but this high angle does not work for me in the clinical setting. I have changed the angle to be quite low when I penetrate the skin on patients. It works well for me since I avoid penetrating right through the vein (Student 5).*

The students assessed their own limitations; namely, that a failed vein cannulation should not be a reason for exposing patients to unnecessary discomfort and pain.

*As a rule, I try twice. If I cannot master it in two attempts, I let someone else try (Student 6).*

The students made professional assessments regarding the size of the vein cannula to be used. These assessments were not necessarily accurate because they were based on the size of the patient's vein and not the purpose of the infusion.

*I believe it is the size of the vein that determines the size of the vein cannula to be used. I consider what to use after I have seen the patient's vein (Student 6).*

## 4 Discussion

This study aimed to impart an understanding of student nurses’ learning opportunities to develop peripheral vein cannulation competency via different learning modalities: latex arms, peers, and patients. In the simulation setting, students could choose to practice peripheral vein cannulation on a latex arm, peer's arm, or both. This implies that the sequence of the students’ peripheral vein cannulation attempts was random and not organised, based on a pedagogical way of thinking.

Although practicing peripheral vein cannulation on a human arm is more complex than that on a latex arm (Bridge et al., 2022; Ravik et al., 2015), the simulation-based peripheral vein cannulation practice did not range from less to more complex for all students. This pedagogy is not consistent with the conventional perception of the organisation of learning, which emphasises learning as a developmental trajectory. Once the less challenging parts of the task are learned, they can be integrated into more challenging parts (Bruner, 2006). However, an accepted simulation-based pedagogy for practicing peripheral vein cannulation has not been thoroughly explored in nursing education (Arslan, 2021; Hilleren et al., 2022). Our findings suggested the use of sequencing in the simulation setting; practicing on a latex arm is necessary before practicing on a peer's arm. Familiarity with the skill's steps and equipment on the latex arm provides fundamental knowledge before moving on to practice cannulation on a peer's arm and consequently, promotes an active and constructive learning process (Bruner, 2006).

A failure to control the sequence from the less complex to the more complex in the simulation-based peripheral vein cannulation skill learning resulted in different student prerequisites when practicing the skill on patients in the clinical setting. Students were more uncertain about the cannulation and hesitant to perform the skill on a patient's arm if they had practiced only on a latex arm. Students who had practiced on a peer's arm rapidly progressed to practice on patients, probably because of the experience of a connection between the learning and application settings (Marton, 2006). Although patients had medical disorders, unlike peers, the differences between these training modalities were much less than the differences between a latex and human arm, thereby supporting the transfer of previous knowledge. For both training modalities on a human arm, there was a similar feature. If the cannula was improperly inserted before stylet withdrawal, the students had to terminate the cannulation (Ravik et al., 2015; Ravik et al., 2017a; Ravik et al., 2017b; Ravik and Bjørk, 2021). Conversely, students could continue to practice on the latex arm even if the vein cannula was improperly inserted (Ravik et al., 2015). Therefore, practicing peripheral vein cannulation on a latex arm alone may have rendered it challenging for some students to be active and immersed in the clinical setting, and the differences between the two learning settings became considerably more extensive.

Student nurses experienced that different learning modalities during peripheral vein cannulation practice afforded varied levels of realism and learning experiences. Realism is the degree to which a training modality or simulation imitates the state and behaviour of real-world objectives and characteristics; i.e. the individual's experiences of the ‘look-and-feel’ of the training modalities or situations (Lioce, 2020; Schutte et al., 2018). Several students mentioned the lack of both anatomical and physiological realism during practice with a latex arm, which resulted in inaccuracy or omitting of important steps. Inadequate learning of peripheral vein cannulation skills with latex arms has been reported; a latex arm did not make the student aware of incorrect performances (Ravik et al., 2015) nor of any variability in a person's arm (Bridge et al., 2022). Consequently, a latex arm may not provide appropriate learning opportunities and prepare students to transfer previous learning to actual hospital settings.

Practicing on peers represented a shift in students’ experiences of realism; the training became more serious and authentic. The latex arm contributed to learning about the equipment and steps of peripheral vein cannulation, whereas practice on a peer's arm contributed to a more thorough skill learning because of the presence of real skin and veins with varied visibility and depth. This corresponds to students’ assertion that practice on a peer was a prerequisite for being mentally prepared to practice vein cannulation on a patient's arm. Realism during training was especially evident when the students practiced peripheral vein cannulation on patients. Several of the students’ comments exemplify the challenge of moving from healthy and well-nourished peers’ arms to patients’ arms that were influenced by age, medical disorders, and treatment. Previous studies have emphasised the value of using patients as training modalities for learning (Bell et al., 2009; Ewertsson et al., 2017; Ravik et al., 2017b); practicing peripheral vein cannulation on patients was important to develop an embodied knowledge, where ‘the body knows how to act’, and to experience the complexity of the skill (Ewertsson et al., 2017; Ravik et al., 2017b).

Peripheral vein cannulation practice triggered various emotions in the students, most of which appeared rooted in their insecurity and nervousness related to the invasive aspects of the skill, and apprehensions of hurting the person they practiced on. Moreover, in the students’ narratives, they were aware that their own reactions were intertwined with the emotional reactions, or the lack thereof, in the other person. However, students did not react emotionally when they practiced vein cannulation on a latex arm, as no kind of relationship could be formed with it, indicating that practice on task trainers tends to be more technical rather than emotionally focused (Aebersold, 2018; Hilleren et al., 2022). Conventionally, the simplest manikins are used during training, and they lack features that consider emotional aspects (Aebersold, 2018). Task-driven learning may hinder students’ competency for meeting relational and psychosocial patient needs. For inexperienced nursing students, regulating their emotions when attempting to form relationships with patients in their first clinical placements is often challenging because this issue is seldom considered in the academic setting (McCloughen et al., 2020).

Progression to practice on a peer's arm made it more natural for students to display empathy because communicating with someone who could verbalise and had facial expressions was possible. In addition to being responsible and apprehensive of injuring their peers, students were empathetic and emotionally sensitive when experiencing that peers were equally anxious. To the best of our knowledge, only a few nurse education courses, at least in Europe, permit students to practice invasive skills on their peers. These restrictions may be based in ethical, psychological, or legal reasons; however, a simulated setting can be an advantageous place to explore emotions and reactions that students experience when practicing on patients. The simulated setting is a third learning arena situated between the theory and clinical arenas (Laursen, 2016). It is a safe place to practice, as students do not meet sick and vulnerable patients. This middle position facilitates the integration of theory and practice, and these learning possibilities may be underutilised. In a recent review on practical skill learning in the simulation setting, the learning processes were hardly elucidated; only the effect of different kinds of practice, mostly students’ development of skill and knowledge, were researched (Hilleren et al., 2022).

During placement in the clinical setting, students’ experiences pertaining to the relationships they had formed with patients considerably influenced their own emotional reactions when performing peripheral vein cannulation. Patients’ attitude and involvement in the relationship were the pivotal stimuli that triggered the students’ emotional reactions and their actions. According to the Fundamentals of Care Framework tenets (Kitson et al., 2014), technical skills along with relational and caring skills advance learning. Good communication and rapport with patients eased students’ technical efforts of placing peripheral vein cannulas. Similar to the study findings of Manninen et al. (2014), the students felt that patients could teach them based on their former peripheral vein cannulation experiences; they were accommodating and allowed students to reattempt cannulation after aborted attempts. In a meta-synthesis, (Kaldal et al., 2018, p. 103) found that all studies reported that student nurses experienced a ’pervasive anxiety or fear’ in patient care encounters. In our study, students were apprehensive and anxious when encountering patients who were nervous and unwilling to allow students to perform a peripheral vein cannulation; however, these feelings were not pervasive.

Although this was the first hospital placement and opportunity to practice peripheral vein cannulation on patients, many students exhibited the capacity to offer professional nursing assessments related to their own practice. These assessments were activated by encountering situations that were different (e.g. the need to shift the angle of the cannula to prevent venous perforation) and contrary (e.g. lower limb peripheral vein cannulation involved more risks for the patient than upper limb peripheral vein cannulation) to the knowledge they had learned in the simulation setting. Furthermore, the students’ narratives revealed that they lacked both knowledge and experience to make correct assessments, despite their attempts; for example, when choosing a cannula based on the size of the patient's vein instead of the purpose of the infusion, suggesting an instance of inadequate similarity between the learning situations (García-Expósito et al., 2021).

In accordance with Thorndike's theory of identical elements (Woodworth and Thorndike, 1901), a behaviouristic approach, transfer in learning is based on the similarities between the learning (simulation) and transfer settings. Thus, these similarities may enable students to continue learning in the new setting. However, Marton (2006) emphasised the importance of experiencing differences and similarities in transfer of learning and that repeated practice on a latex arm can get the students only so far toward skill proficiency. According to Marton (2006), ‘Variation is necessary for discernment, and learners must discern critical differences between situations in order to be able to adjust to new situations’. Students’ descriptions of learning are closely linked to their experiences of the differences as they progressed from one learning modality to the next, especially when practicing on patients in the clinical setting.

Owing to shorter hospitalizations, lack of internships, or inadequate supervision, the replacement of clinical learning hours by simulation-based learning has been much debated (Bridge et al., 2022; Hayden et al., 2014). Our findings suggested that this is an unviable solution. Students stated that the clinical setting provided opportunities to learn the complexities of peripheral vein cannulation. Bridge et al. (2022) refuted that simulation-based learning was a replacement for clinical learning experiences because of these complexities. Conversely, Meiers and Russell (2019) contended that practical skill complexities can be learned in the simulation setting by implementing clinical contextual conditions. They suggested the use of unfolding cases that introduced new contextual data throughout a longer period of practicing several skills. This pedagogical approach may broaden the students’ opportunities to incorporate more and different types of knowledge during technical skill learning. However, it does not necessarily improve students’ opportunities to learn the relational and communicative competency that is needed for patient care during practical skill learning in a clinical setting.

### 4.1 Limitations

There was a risk that dominant individuals within the focus groups might influence others’ constructs. The lead researcher was aware of this challenge and orientated all participants to speak freely individually. Data collected in late 2012 were analysed. This is due to the large amount of original data that have been analysed and published continually since 2013. Although these data are rather old, to our knowledge, recent research findings consistent with our perspectives are unavailable in the literature on this topic.

## 5 Conclusions

In the present study, three training modalities (a latex arm, a peer, and a patient) provided students with different learning opportunities in peripheral vein cannulation, and these approaches may possibly supplement each other. Our findings indicate a need to further review peripheral vein cannulation skill learning to enhance the transfer of learning from the simulation setting to the clinical setting; for example, whether patient-contributed factors in this transfer process might provide a better success rate among newly qualified nurses when performing peripheral vein cannulation.

## Other author footnotes

1 Member of the research group Research in Nursing Skills (RiNS), www.rins.dk
  Leader of the research group Clinical Competence in Nursing Education, University of South-Eastern Norway, Norway.
2 Leader of the research group Research in Nursing Skills (RiNS), www.rins.dk

## Contribution of the paper

### What is already known

• Peripheral vein cannulation is a fundamental but challenging skill to learn and master.
• Student nurses learn peripheral vein cannulation in simulation and clinical placement settings.
• Research is limited on student nurses’ learning outcomes when training in peripheral vein cannulation in simulation or clinical placement settings.

### What this paper adds

• We found that training on peripheral vein cannulation in a simulation setting and in clinical placements provided student nurses with different training and learning opportunities.
• A simulation setting does not replace the clinical setting because of the relational and communicative competency that is needed for patient care.
• The realism and complexity of peripheral vein cannulation was particularly experienced in the clinical setting.

## Funding sources

This research did not receive any specific grant from funding agencies in the public, commercial or not-for-profit sectors.

## Authors’ contributions

Both authors conceptualized the study, developed the interview guides, conducted the data analysis, and drafted and revised the manuscript. Both authors approved the manuscript. The first author conducted the data collection.

## Data availability statement

To access the data in this study, please contact the corresponding author.

## Declaration of Competing Interest

None.
